# Lead Generation and Optimization Based on Protein-Ligand Complementarity

**DOI:** 10.3390/molecules15064382

**Published:** 2010-06-17

**Authors:** Koji Ogata, Tetsu Isomura, Shinji Kawata, Hiroshi Yamashita, Hideo Kubodera, Shoshana J. Wodak

**Affiliations:** 1 Centre for Computational Biology, The Hospital for Sick Children, 555 University Avenue, Toronto, Ontario M5G 1X8, Canada; 2 Advanced Medical Research Laboratories, Mitsubishi Tanabe Pharma Corporation, 1000, Kamoshida-cho, Aoba-ku, Yokohama, Kanagawa 227-0033, Japan; 3 Medicinal Chemistry Research Laboratories, Mitsubishi Tanabe Pharma Corporation, 1000, Kamoshida-cho, Aoba-ku, Yokohama, Kanagawa 227-0033, Japan; 4 Department of Biochemistry, University of Toronto, Toronto, Canada; 5 Department of Molecular Genetics, University of Toronto, Toronto, Canada

**Keywords:** lock-and-key problem, computational structure-based drug design, lead generation, lead optimization

## Abstract

This work proposes a computational procedure for structure-based lead generation and optimization, which relies on the complementarity of the protein-ligand interactions. This procedure takes as input the known structure of a protein-ligand complex. Retaining the positions of the ligand heavy atoms in the protein binding site it designs structurally similar compounds considering all possible combinations of atomic species (N, C, O, CH_3,_ NH, *etc*). Compounds are ranked based on a score which incorporates energetic contributions evaluated using molecular mechanics force fields. This procedure was used to design new inhibitor molecules for three serine/threonine protein kinases (p38 MAP kinase, p42 MAP kinase (ERK2), and c-Jun N-terminal kinase 3 (JNK3)). For each enzyme, the calculations produce a set of potential inhibitors whose scores are in agreement with IC50 data and Ki values. Furthermore, the native ligands for each protein target, scored within the five top-ranking compounds predicted by our method, one of the top-ranking compounds predicted to inhibit JNK3 was synthesized and his inhibitory activity confirmed against ATP hydrolysis. Our computational procedure is therefore deemed to be a useful tool for generating chemically diverse molecules active against known target proteins.

## 1. Introduction

When a high-resolution structure of a target protein is known, computational structure-based drug design is an efficient and effective methodology for the identification and further optimization of hit compounds in order to generate lead compounds. Several studies [1,2,3] have reported the successful identification of hit molecules by *in silico* screening of large compound databases using software packages such as DOCK [4], GOLD [5,6] and eHiTS [7]. In many cases, however, identified hits exhibit weak biological activity and poor Adsorption, Distribution, Metabolism, Excretion and Toxicity (ADMET) properties, making them unsuitable scaffolds for further optimization. Consequently, new strategies have to be developed in order to derive molecules with better biological properties from such hits.

Standard hit compound optimization approaches involve the addition, replacement or removal of chemical groups within the hit molecule. However, enhancing the biological activity of the hit often requires a more drastic modification of the core molecular skeleton [8]. *De novo *design [9,10,11,12,13] and scaffold hopping techniques [14,15,16,17,18,19,20,21,22,23,24,25,26] are examples of methods that involve such modifications. The general assumption underlying these methods is that compounds with similar geometries will interact in a similar manner with the target protein and therefore, will show similar or improved inhibitory activity. This assumption is based on the lock-and-key model for protein-ligand interactions [27] and most of the methods are based in making changes in the native moiety of the ligand scaffolds and their geometries.

In this paper, we report the application of a computational method for structure-based ligand optimization to three serine/threonine protein kinase systems: p38 MAP kinase, p42 MAP Kinase (Erk2), and c-Jun N-terminal kinase 3 (JNK3). Our method uses as starting point, the atomic coordinates of the protein-ligand complex, determined by X-ray Crystallography, NMR, or modeling techniques [28]. A large number of compound candidates are generated by replacing the atoms of the native ligand with different substituents, keeping fixed the atomic positions of its core skeleton. Each new compound replaces the native ligand in the protein active site complementarity and the new protein-ligand complex is scored in order to rank lead candidates. The top ranking compounds are selected for further analysis. The innovation of our approach lies in the way in which the library of compound candidates is generated as well as in the rapid identification of the chemical groups (and combinations thereof) that is likely to enhance biological activity. Our method identifies the native ligands among the top five hit compounds for the three serine/threonine Kinases analyzed here. In addition, the five top ranking compounds exhibit significant IC50 levels against ATPase activity [29,30]. In the JNK3 systems, a compound from the 10 top ranking candidates was chemically synthesized and IC50 measurements showed inhibitory activity against ATP hydrolysis. These results suggest that our method can be useful in the identification and generation of lead compounds as drug candidates.

## 2. Results and Discussion

**Figure 1:**
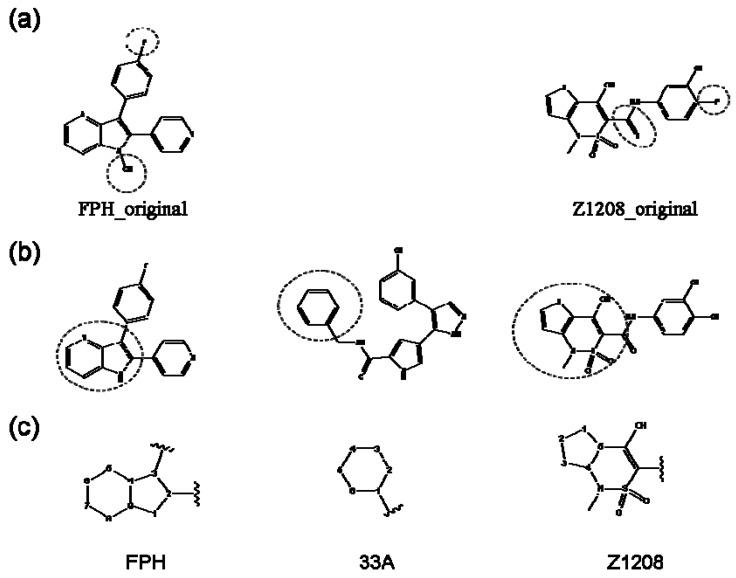
Chemical structures of the native compounds and modifications made to create the input files for our calculations (see text for details). The portion surrounded by the dotted circle was replaced as indicated in the text, and position numbers are displayed for illustrative purposes.

Our approach was tested using three *serine/threonine protein kinases* as targets. The X-ray protein-ligand complex structures used in this study were: p38 MAP kinase/3-(4-fluorophenyl)-2-pyridin- 4-yl-1*H*-pyrrolo[3,2-b]pyridine-1-ol (FPH) complex, p42 MAP Kinase (Erk2)/*N*-benzyl-4-[4-(3-chlorophenyl)-1*H*-pyrazol-3-yl]-1*H*-pyrole-2-carboxamide (33A) complex, and c-Jun N-terminal kinase 3 (JNK3)/*N*-(3,4-dichlorophenyl)-4-hydroxy-1-methyl-2,2-dioxo- 1,2-dihydro-2lamda~6~-thieno[3,2-c][1,2]thiazine-3-carboxamide (in house code Z1208) complex obtained from the Protein Data Bank (PDB) [31] (see Table 1). In preparing the input structures for our calculations, the FPH and Z1208 were modified as mentioned in Experimental section (Figure 1).

**Table 1:** Proteins and ligands used in this study.

Protein names	PDB entry	Name of ligands	PDB entry	MW
Map kinase P38	1oz1	3-(4-FLUOROPHENYL)-2-PYRIDIN-4-YL-1 *H*-PYRROLO- [3,2- B]PYRIDIN-1-OL	FPH ^1^	305.306
Map kinase ERK2	2oji	*N*-BENZYL-4-[4-(3-CHLOROPHENYL)-1*H*-PYRAZOL-3-YL]- 1*H*-PYRROLE-2-CARBOXAMIDE	33A	376.839
c-Jun N-terminal kinase 3 (JNK3)	2ok1	*N*-BENZYL-4-[4-(3-CHLOROPHENYL)-1*H*-PYRAZOL-3- YL]-1*H*-PYRROLE-2-CARBOXAMIDE	33A	376.839
c-Jun N-terminal kinase 3 (JNK3)	NA	*N*-(3,4-DICHLOROPHENYL)-4-HYDROXY-1-METHYL-2,2-DIOXO-1,2-DIHYDRO-2LAMBDA~6~-THIENO[3,2-c][1,2]THIAZINE-3-CARBOXAMIDE	Z1208 ^2^	405.278

^1^ The prediction was used for a modified compound in which the OH group in pyrrolo[3,2-b]pyridine ring was replaced by a hydrogen; ^2 ^The tertiary structure was determined by X-ray crystallography at ZOEGENE Corp.

### 2.1. Redesign of the FPH ligand

**Figure 2:**
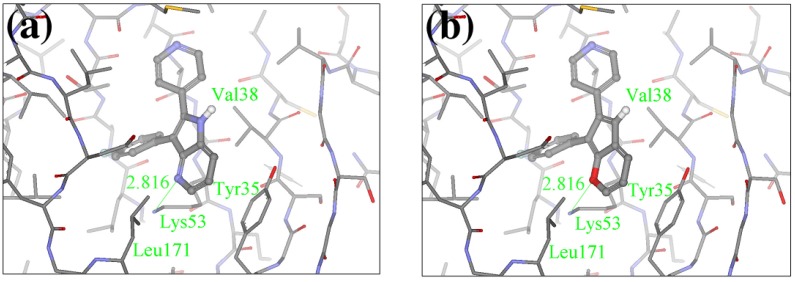
FPH-p38 MAN kinase complex analysis. Binding conformations of (a) native FPH, and (b) the top scoring compound in our calculations.

For the FPH-p38 MAP kinase complex, 230 candidate molecules were obtained by our calculations after the various filters have been applied. Our results show that the preferred atoms for position 5 in the 6-membered ring (see Figure 1c) were either –O– or –N=, to enable hydrogen bond formation with the Nζ of Lys53 (numbering as in the PDB entry 1oz1) (see Figure 2a). The same hydrogen bond is observed in the original structures of the p38-FPH complex [30]. In order to maintain this hydrogen bond, but also satisfy the bond orders of the 5- and 6- condensed membered rings, specific combinations of the atomic species containing single and double bonds are required. For example, if an –O– group is assigned at position 5 (see Table 2), other positions in the molecule could be occupied by either sp2 atoms (ex. compound 1, 5 and 8), or the combination of two sp3 atoms and six sp2 atoms (ex. compound 3, 4). If an –N= group is assigned at position 5, the other positions in the molecule could be occupied by one sp3 atom and seven sp2 atoms (for example, compounds 2, 6). Detailed analysis of the p38-FPH binding site, revealed that the condensed FPH ring formed hydrophobic interactions with Val38, Leu171, and the aromatic ring of Tyr35 (see Figure 2). These interactions are maintained by some of the compounds described earlier in this paragraph. In summary, our method assigned a hydrogen bond acceptor groups to position 5 and hydrophobic groups to the other positions in the condensed rings thereby satisfying binding features observed in the original crystal structures. 

**Table 2:** The 10 highest scoring FPH substitutions for the FPH-p38 MAN kinase complex.

Ranking	Compounds	Score (Kcal/mol)	Ranking	Compounds	Score (Kcal/mol)
1	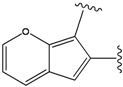	-38.536	6	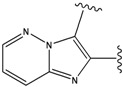	-37.916
2	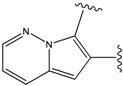	-38.51	7	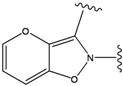	-37.834
3	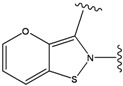	-38.474	8	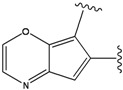	-37.602
4	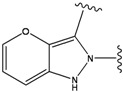	-38.036	9	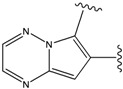	-37.577
5	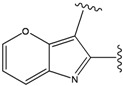	-37.941	10	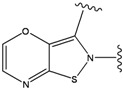	-37.541

We find that the scores computed by our method for the four compounds 9, 22, 24, and 35, correlate well with the experimental IC50 values (see Table 3). In addition, the suggested top-scoring compound (compound 9), also features the lowest IC50 among the six compounds. In this candidate, the geometry of native FPH (see Figure 1) was modified by replacing a hydroxyl group with a hydrogen atom, as mentioned below (Section 3.2). Having introduced this modification, we might expect that the binding mode of the modified compound would differ somewhat from the native ligand. However, in the current version of our method such alternative binding modes are not considered. Without experimental information on the binding mode of compound 9, it is therefore difficult to accurately evaluate at this point the actual predictive power of our calculations.

**Table 3:** Calculated scores *versus* IC_50_ values for FPH substituents.

Compounds	Substitution	Ranking	Score (Kcal/mol)	IC50 (nM)^1^
9	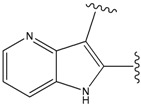	29	-36.501	6.5
15	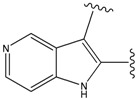	153	40.118	3100
19a	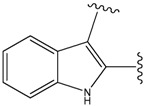	156	40.214	1800
22	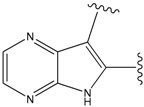	56	-35.568	53
24	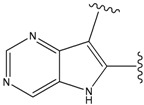	66	-34.414	895
35	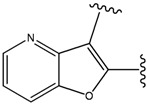	34	-36.299	86

^1^ IC_50_ values are from Trejo *et al * [30].

We also find that the scores of low ranking compounds tend to be inconsistent with their IC_50_ values. For example, compounds 15 and 19a, whose ranks are 153 and 156 respectively, have IC_50_ values of 3,100 nM and 1,800 nM (see Table 3). However, despite the discrepancy in these values, our calculations indicate the correct binding trend since the scores of both compounds, are positive, which is indicative of unfavorable binding energies, in agreement with their experimental IC_50_ values. Detailed analysis of the generated structures suggests that this unfavorable score can be explained by the lack of hydrogen bond capability of position 5 in these compounds, as stated above (see Figure 2).

### 2.2. Redesign of the 33A scaffold to optimize ERK2 binding

**Figure 3:**
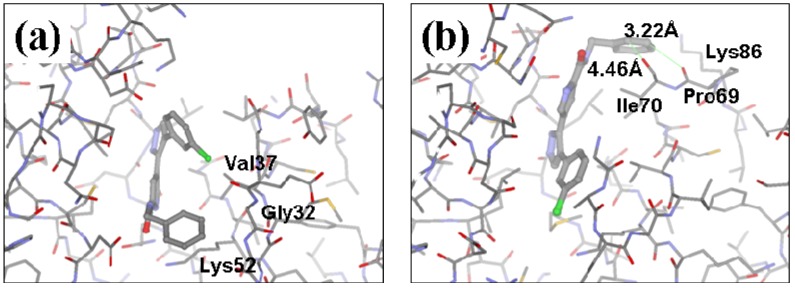
Structures of the complexes of 33A with (a) ERK2 and (b) JNK3, respectively. Both the proteins and the ligand are displayed using stick models, with the ligand shown using thicker bonds. Comparison of panels (a) and (b) illustrate the difference in orientation of 33A in the two structurally aligned proteins.

The ATP binding sites in ERK2 and JNK3 exhibit different chemical compositions and in particular different ratios of hydrophilic *versus* hydrophobic residues (see Figure 3a,b). Consequently, ligand 33A displays different binding orientations in ERK2 and JNK3, with the chlorobenzene moieties oriented in opposite directions. Analysis of the ERK2 complex structure revealed contacts between positions 3 and 5 of the aromatic ring of the ligand with hydrophobic groups of the protein (Ca in Gly32, Cg2 in Val37 and Cd in Lys52) (see Figure 3a). Hydrophobic groups were hence the preferred substituents for these two ring positions. Furthermore, position 4 of the aromatic ring showed interactions with the carbonyl oxygen of Ala33, suggesting hydrogen bond donor groups as preferred subtituents for this position.

Our calculations yielded 23 different substituents for the A33 ring scaffold (Figure 1c), with benzene moiety having the top score for the ligands-ERK2 complexes (see Table 4). 

**Table 4:** Five best scoring substitutions for the complexes ERK2 and JNK3 kinases with A33.

ERK2				JNK3			
Ranking	Substitutions	Score	Ki, uM^1^	Ranking	Substitutions	Score	Ki, uM^1^
1	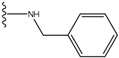	-28.016	0.086	1	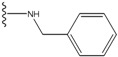	-43.488	0.55
2	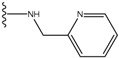	-27.643	---	2	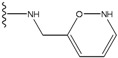	-42.738	---
3	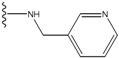	-27.342	0.23	3	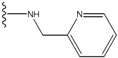	-42.557	---
4	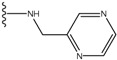	-26.969	---	4	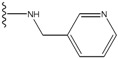	-42.117	ND^2^
5	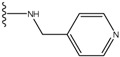	-25.62	0.16	5	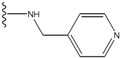	-41.903	ND^2^

^1^ Ki values are from the article by Aronov *et al*. [29]; ^2^ ND (Not determined). Authors reported that compound 4 was 3-fold less active than 1 and compound 5 was 2-fold less active.

These results are in agreement with the inhibitory activity (lowest *K_i_* value) previously reported [29] and can be explained by the hydrophobic interactions between the aromatic ring and the active site protein residues. The substitution of a –CH residue by –N= decreases the hydrophobic interactions and may explain the lower score values of the other designed compounds. The exception to this rule is the 5^th^ ranking compound where the addition of –N= residue to position 4 in the aromatic ring increased the repulsive energy (decreasing the overall score) due to the proximity of this substituent to the carbonyl oxygen of the residue Ala33. This effect is enhanced by the fact that our software is using a fixed geometry approximation. Two approaches are currently being developed to improve this methodology: (a) consideration of different compound conformations, and (b) relaxation of the protein-ligand complex in order to relieve any residual strain. 

### 2.3. Redesign of the 33A scaffold to optimize JNK3 binding

Analysis of the 3D-structure of the 33A-JNK3 complex revealed that the ring scaffold to be modified interacts with Lys68, Pro69 and Ile70 (see Figure 3b). The ring positions 2 and 3 are close to the carbonyl oxygen atoms of the protein residue Pro69 and Ile70, while the rest of the ring atoms are surrounded by hydrophobic residues. Therefore, positions 2 and 3 were assigned positively charged substituents, whereas hydrophobic substituents were assigned for the remaining ring positions.

Subject to these constraints, our procedure sampled a total of 23 substitutions for the 6-membered 33A ring (Figure 1c). Here too, our results revealed benzene moiety to be the best ranking compound in agreement with the experimental data [29], with a similar rationale as for the ERK2. Our second “best compound” contains an –O– at ring position 2 and an –NH– group at ring position 3. The somewhat lower score of this compound is due to close polar contact with backbone atoms of the protein. This score is driven by the stabilizing energy from the proximity between ligand –NH- to the carbonyl oxygen O of Pro69 (3.23 Å), and the repulsive energy for the interaction between the ligand –O– at position 2 with the carbonyl oxygen of Ile70 (4.46 Å). 

The compounds with 2-, 3- and 4-pyridine moieties were ranked at 3rd, 4th and 5th positions, respectively. With respect to the calculated “best compound”, these scores can be rationalized in the same way as in the case of ERK2 case, by a decrease in hydrophobic interactions. However, the authors of the experimental paper [29] found that compounds with 3- and 4-pyridine moieties do not bind to JNK3, which underscores the difficulty in discriminating between compounds that bind from those that do not bind, based on our computed scores alone. 

### 2.4. Redesign of the Z1208 scaffold bound to JNK3.

**Figure 4:**
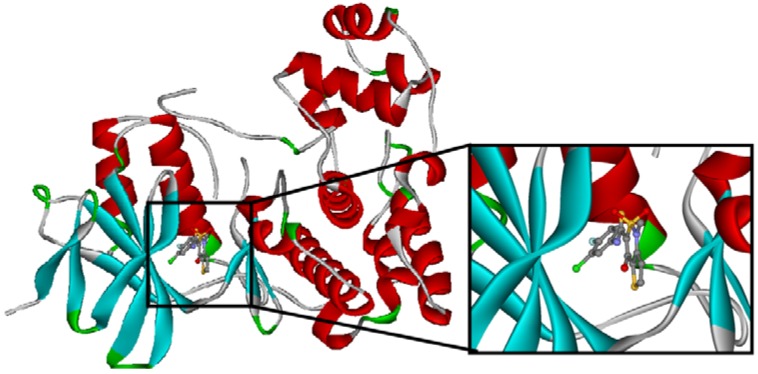
Binding site of the native Z1208 JNK3 complex.

The crystal structure of the native Z1208 with JNK3 complex suggests that the ligand is tightly bound to the ATP binding pocket of the protein (Figure 4). 

**Figure 5:**
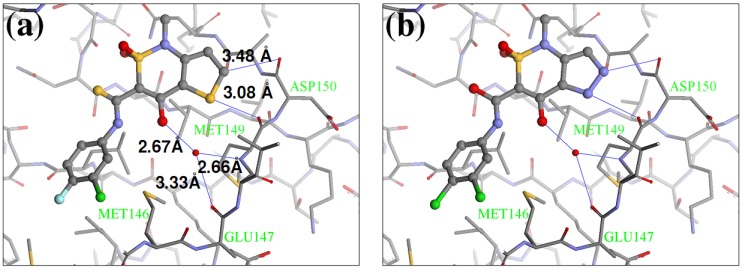
Conformations of (a) native Z1208 and (b) Z1208_8 binding to JNK3.

One water molecule appears in the binding site forming hydrogen bonds with the protein backbone (carbonyl oxygen of the Glu147 and the amide nitrogen of Met149) and the hydroxyl group of the condensed ring in the native Z1208 (Figure 5a). Analysis of JNK3-ligand complexes in the PDB show that hydrogen bond interactions between the ligand and the backbone atoms of Glu147 and/or Met149 are common, but the water molecule is not present in all the structures. Therefore for the purpose of this study the water molecule was removed. Furthermore, in the binding site, the 3-chloro-4-fluoro-phenyl moiety of the native Z1208 ligand is positioned near Met146 of JNK3 and surrounded by other hydrophobic residues (Figure 4). According to Scapin et al. [32], JNK3 complexes with imidazole-pyrimidines (PDB-codes: 1qmn and 1qmq) that have halogen-phenyl moieties, feature different conformations of the Met146 side chain than in the complex with AMP-PNP (PDB-code 1jnk). We found that the structure of JNK3 protein in the complex with Z1208_original, superimposed well onto those of the JNK3 proteins in 1qmn and 1qmq. In addition the Met146 adopts a similar conformation on all three complexes.

Our calculations generated 64 designed compounds. The top 10 ranking compounds are displayed in Table 5. Compounds with the best and the second best scores contain one –NH– group in different positions of the 5-membered ring. The addition of a second substituent in the same ring of the molecule (–NH– and –N=), produced only a slightly higher score (compound 8 in Table 5). Visual inspection of the modeled ligand-protein active site reveals that oxygen atoms in Met149 and Asp150 make contacts in the 5-membered ring, and those atoms are engaged in repulsive interactions when the negatively charged –N= groups are introduced (Figure 5a). Hence replacements which combine sulfur or carbon atoms with the –NH– group is preferable to those of two –N= groups.

**Table 5:** Ten best scoring substitutions of Z1208 bund to JNK3 and their IC50 values.

Ranking	Substitutions	Score	IC50 (uM)	Ranking	Substitutions	Score	IC50 (uM)
1	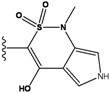	-37.376	NA	6	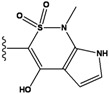	-35.815	NA
2	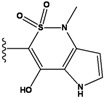	-36.783	NA	7	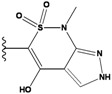	-35.783	NA
3	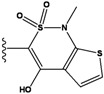	-36.6	NA	8	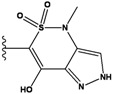	-35.569	
4	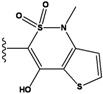	-36.285		9	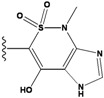	-35.189	NA
5	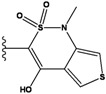	-36.145	NA	10	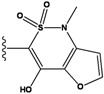	-35.175	NA

We see that the native compound is ranked 4th in this list, but its score is only slightly worse than the predicted best-scoring ligand. Unfortunately, the chemical synthesis of all three best ranking compounds was very difficult with current techniques, demonstrating that the filters used in the pre-calculation steps were not sufficient to remove all the unsynthesizable compounds and hence further improvements of the various filters used in our procedure are required. Nevertheless, it is quite encouraging that the native ligand was the top ranking synthesizable compound.

### 2.5. Discussion

#### Additional validation of our method by synthesis of one molecule

An additional validation method step was carried out by synthesizing in the laboratory one of the top-ranking newly designed inhibitors. The chosen compound was Z1208-8, ranked 8th in Table 5, and its inhibitory activity was measured against ATP hydrolysis by JNK3. This compounds was selected because it was easier to synthesize compared to other higher ranked compounds in the same list. 

The biological assay showed that Z1208-8 was able to inhibit ATP binding to JNK3 with an IC_50_ 62.9 μM, representing a six fold poorer activity than the native ligand (Z1208, 9.6 μM). The analysis of the 3D structure of the X1208-8/JNK3 complex indicated that the two major contributions to the overall energy score are: 1) the NH group in the 5-membered ring forming a hydrogen bond with the carbonyl oxygen of Asp150, which would favor binding, and 2) a close contact of the –N= group in the 5-membered ring with the backbone oxygen of Met149 (Figure 5b), which would disfavor binding. The second contribution is higher in Z1208-8 higher than in the native compounds, suggesting an explanation to the higher IC_50_ value observed in the new inhibitor. 

Our method is thus capable of designing new compounds with inhibitor activity against the target enzyme, but their activity can be lower than that of the starting hit compound. We expect however, that several cycles of rational design using our method and experimental analysis of the top ranking candidates should lead not only to compounds with different chemical properties from those of the original molecule, but also to those with a strong or stronger inhibitory activity.

This paper has shown that our computational method is effective in a scaffold-based redesign of kinase inhibitors FPH, 33A and Z1208. For a defined scaffold and keeping fixed the “geometry” of its core skeleton, our method was capable of sampling a large number of different substituents providing a set of compounds with potential inhibitory activity against the protein targets. Compounds were ranked based on an energy function, and in all the cases native inhibitors were identified in the 5 top ranking compounds. Validation of our method was performed by comparing the scores of designed molecules with empirical data on their inhibitory activity (IC50 and Ki values). In the JNK3 inhibitor design study, one of the top ranked compounds was synthesized and its inhibitory activity was confirmed experimentally. 

Future developments will address the following outstanding limitations of our method: 1) more effective filters to remove compounds difficult or impossible to synthesize, 2) improve the scoring function to enhance compound ranking accuracy, and 3) take into account protein and ligand conformational flexibility and different ligand poses in the protein active site. With the introduction of these improvements, our computational approach holds the promise of becoming a useful tool for lead optimization. 

## 3. Experimental

### 3.1. Lead optimization procedure

The lead/hit optimization procedure used in this study was previously reported by Ogata *et al* [33] and is only briefly summarized here. The first step consists in extracting the atomic coordinates of the ligand’s heavy atoms (referred to as ‘geometry’) from a high resolution structure of the protein-ligand complex. The geometry is then divided into fragments which are grouped into three partial structures types: *rings*, *linkers *(defined as the fragments that connect rings), and *terminals* (defined as other types of fragments). In addition, all the atoms in the geometry are classified according to their bond order types (sp3, sp2, etc ) and atomic species (CH_3_, CH_2_, CH, NH_2_, NH etc). For example, consider the geometry X···Y···Z, in which X, Y, and Z represent the atoms in the geometry and ‘···’ is a generic representation of the bonds connecting the atoms. Replacing Y with a “=CH–” generates a chemically incomplete compound X=CH–Z for which “=” and “–” indicate a double and a single bond, respectively. Then, X should be assigned to an atomic species linked through a double bond to Y (ex. O= or CH2=). Similarly, Z should be assigned to an atomic species capable of linking to Y through a single bond (ex. –CH_3_ or –NH_2_). By assembling all possible combinations of these atomic species, four compounds are obtained: O=CH–CH_3_, O=CH–NH_2_, CH_2_=CH–CH_3_, and CH_2_=CH–NH_2_. For the work presented in this paper, eighteen atomic species were used (see Table 6). 

**Table 6:** Atomic Chemotypes used in this study.

Atom groups	Bond type	No. of bonds	No. of hydrogens	Atom groups	Bond type	No. of bonds	No. of hydrogens
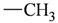	sp3	4	3	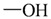	sp3	2	1
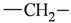	sp3	4	2	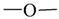	all	2	0
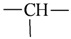	sp3	4	1	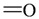	all	2	0
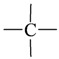	sp3	4	0				
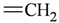	sp2	4	2	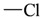	sp3	1	0
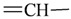	sp2	4	1				
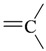	sp2	4	0				
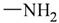	sp3	3	2				
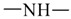	all	3	1	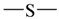	all	2	0
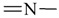	sp2	3	0	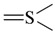	all	4	0
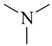	all	3	0	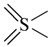	all	6	0

After assigning all possible combinations of atomic species to the native ligand’s core coordinates, all the partial structures are considered and bond order requirements are satisfied thereby generating the hit compound database. Compounds in the newly generated database have similar core geometries as the native ligand and each atomic position satisfies different chemically meaningful combinations.

**Figure 6:**
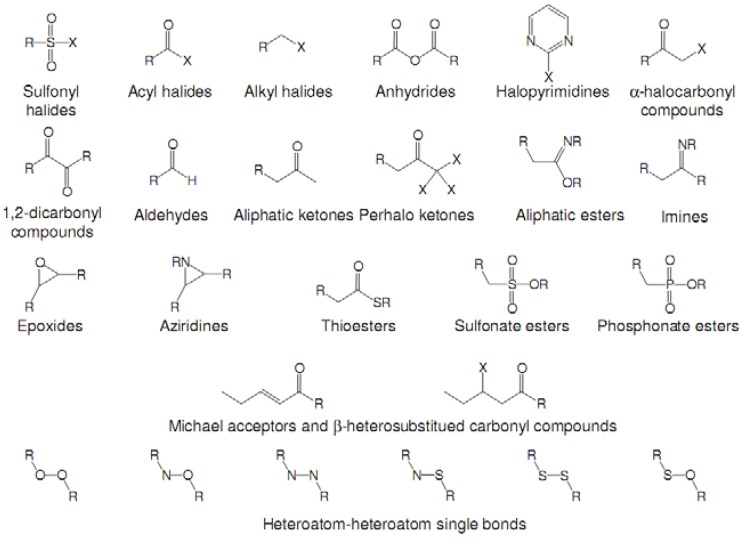
Nonleadlikness filter. The substituent types were extracted from Rishton work [34]. The electrophilic functional groups shown here are the most common protein-reactive covalent-acting false positives in biochemical assays. Compounds with substituents shown in this figure were removed from our results.

In a second step, compounds from the newly generated database are subjected to two filters. The Rishton nonleadlikeness filter (to remove undesirable functional groups, see Figure 6) [34] and the Lipinski’s rule of five (compounds with more than five hydrogen-bond donors, more than 10 hydrogen-bond acceptors, molecular mass greater than 500 Da, logP values greater than 5, or more than 10 rotatable bonds are not desirable for orally active drugs) [35]. From the remaining list of compounds, molecules with ring(s) and condensed ring structures were selected because known hits for the three target kinase proteins contain such structures. These molecules were treated as the final list of lead candidates and ranked based on a scoring function, *Score,* evaluated for the protein-ligand complex. The scoring function comprises four empirical energy terms:

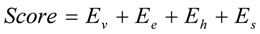

where, *E_v_* is the van deer Waals interaction energy, *E_e_* the electrostatic interaction energy, *E_h_* the hydrogen bond energy, and *E_s_* the solvation energy.

*E_v_* and *E_e_* were obtained using the AMBER force field with the GAFF parameter set. [36] *E_h_* was defined as:

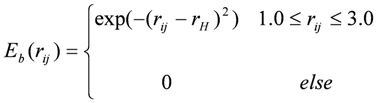
,
where *r_H_* is the distance between the hydrogen and the heavy atom (H^…^X, set at 2.0 Å for this study). 

*E_s _*was computed for the bound and unbound states of the ligand, protein and the complex as:

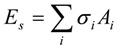
,
where *A_i_* and *σ_i_* are the solvent-accessible surface area and the proportionality factor for the solvent-accessible surface area of atom *i*, respectively [37]. The free energy of 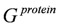
and
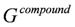
were calculated in the same manner. This method has been designed to provide a ranked list of compounds with better “drug-type” properties (more stable, druglikeness and synthesizable compounds) than other approaches.

### 3.2. Application to serine/threonine protein kinases

Our approach was tested using three *serine/threonine protein kinases* as targets. The X-ray protein-ligand complex structures used in this study were: p38 MAP kinase/ 3-(4-fluorophenyl)-2-pyridin-4-yl-1*H*-pyrrolo[3,2-b]pyridine-1-ol (**FPH**) complex, p42 MAP Kinase (Erk2)/*N*-benzyl-4-[4-(3-chlorophenyl)-1*H*-pyrazol-3-yl]-1*H*-pyrole-2-carboxamide (**33A**) complex, and c-Jun N-terminal kinase 3 (JNK3)/*N*-(3,4-dichlorophenyl)-4-hydroxy-1-methyl-2,2-dioxo- 1,2-dihydro-2lamda~6~-thieno[3,2-c][1,2]thiazine-3-carboxamide (in house code **Z1208**) complex obtained from the Protein Data Bank (PDB) [31] (see Table 2). The three structures display different ligand binding modes, and feature differences in the electrostatic potentials at the ATP-binding site [29,30,38,39,40]. In addition, inhibitory activity against the target proteins has been reported for series of compounds. These compounds were derived by small modifications (changing or adding substituents) of the native ligand structures and atom types [29,30,38]. We used this data to validate the results of our calculations, which involves potential compound candidates with larger structural and chemical differences than the original authors considered in their study.

In preparing the input structures for our calculations, the following steps were performed (see Figure 1): 1) all water molecules were removed from the original complexes’ PDB files; 2) In FPH, the hydroxyl group attached to the 5- and 6-membered condensed ring was replaced by a hydrogen atom because the modified compound has a larger number of similar compounds with experimentally demonstrated inhibitory activity than the native compound; 3) In FPH and Z1208, all the fluorine atoms were replaced by chlorine atoms as this replacement made the chemical synthesis easier and; 4) the thioamide group in Z1208 was replaced by an amide group for the same reason.

In JNK3, two ligands were used to create the input structure: the native Z1208 and a derivative of Z1208 that acts as an ATP hydrolysis inhibitor. The experimental data used for this analysis were the in-house X-ray crystal structure (2.1 Å resolution and R-factor= 24.6%, see Figure 4) and the IC50 values of 22.8 μM and 9.6 μM for native Z1208 and Z1208-derived ATP hydrolysis inhibitor respectively.

### 3.3. Inhibition assay

To measure the inhibitory activity of the Z1208, we used the following assay system: adenosine triphosphate (ATP), phosphoenolpyruvate (PEP), nicotinamide adenine dinucleotide (NADH), and a solution mixture of pyruvate kinase and L-lactate dehydrogenase (PK-L-LDH) were purchased from Roche Diagnostics. Other reagents were purchased from Sigma-Aldrich. JNK3 was expressed and purified by the method of Xie *et al.* [41]. After a purification step, JNK3 was activated by GST-fused MKK7 and further purified against a glutathione-fixed column. Inhibitory activity was estimated by detecting the inhibition of ATP hydrolysis reaction monitored by the coupled reaction of NADH oxidation; a slightly modified method of Xie *et al.* Experimental conditions were: 100 nM JNK3 in 50 mM Hepes, pH 7.6, 10 mM MgCl_2_, 1 mM NADH, 90 mg/mL PK, 30 mg/mL L-LDH, 2 mM PEP, 200 mM ATP, and each concentration of compound under 1% DMSO. The conversion of NADH was measured by kinetic monitoring with SpectraMax 190 (Molecular Devices).

### 3.4. Synthesis of Z1208-8

**Scheme 1:**
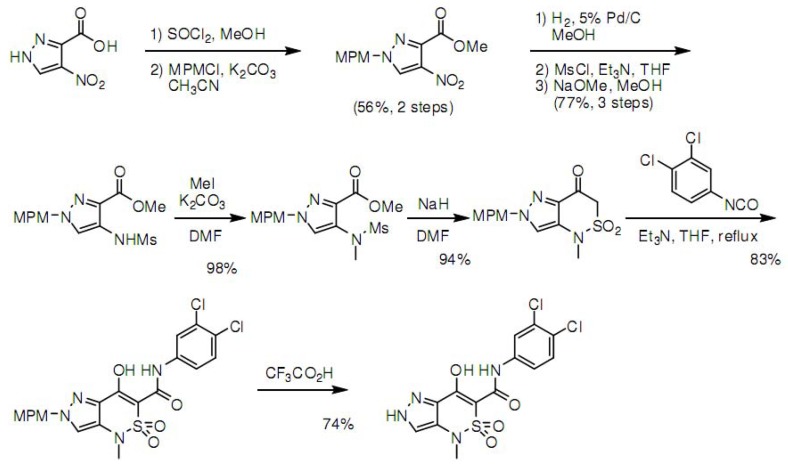
Steps of the chemical synthesis of Z1208-8.

Z1208-8 synthetic path is displayed in Scheme 1 [42,43,44,45].
